# Influence of leukotriene gene polymorphisms on chronic rhinosinusitis

**DOI:** 10.1186/1471-2350-9-21

**Published:** 2008-03-26

**Authors:** Hasan Al-Shemari, Yohan Bossé, Thomas J Hudson, Myrna Cabaluna, Melanie Duval, Mathieu Lemire, Sophie Vallee-Smedja, Saul Frenkiel, Martin Desrosiers

**Affiliations:** 1 Department of Otolaryngology-Head and Neck Surgery, McGill University, Montreal, QC, Canada; 2 McGill University and Genome Quebec Innovation Centre, Montreal, QC, Canada; 3 Centre de Recherche, Hôpital Laval, Institut Universitaire de Cardiologie et de Pneumologie de l'Université Laval, Québec, QC, Canada; 4 Laval University Hospital Research Center (CRCHUL), Quebec, QC, Canada; 5 Ontario Institute for Cancer Research, Toronto, ON, Canada; 6 Department of Otolaryngology-Head and Neck Surgery, Montreal University, Montreal, QC, Canada

## Abstract

**Background:**

Chronic rhinosinusitis (CRS) is increasingly viewed as an inflammatory condition of the sinonasal mucosa interacting with bacteria and/or fungi. However, factors conferring susceptibility to disease remain unknown. Advances in genomics offer powerful tools to explore this disorder. The goal of this study was to evaluate the effect of single nucleotide polymorphisms (SNP) on CRS in a panel of genes related to cysteinyl leukotriene metabolism.

**Methods:**

Severe cases of CRS and postal code match controls were recruited prospectively. A total of 206 cases and 200 controls were available for the present study. Using a candidate gene approach, five genes related to cysteinyl leukotriene metabolism were assessed. For each gene, we selected the maximally informative set of common SNPs (tagSNPs) using the European-derived (CEU) HapMap dataset. These SNPs are in arachidonate 5-lipoxygenase (ALOX5), arachidonate 5-lipoxygenase-activating protein (ALOX5AP), leukotriene C4 synthase (LTC4S), cysteinyl leukotriene receptor 1 (CYSLTR1) and cysteinyl leukotriene receptor 2 (CYSLTR2) genes.

**Results:**

A total of 59 SNPs were genotyped to capture the common genetic variations within these genes. Three SNPs located within the ALOX5, CYSLTR1 and ALOX5AP genes reached the nominal p-value threshold (p < 0.05) for association with CRS. However, none of these SNPs resist multiple testing adjustment.

**Conclusion:**

While these initial results do not support that polymorphsims in genes assessed involved in the leukotriene pathways are contributing to the pathogenesis of CRS, this initial study was not powered to detect polymorphisms with relative risk of 2.0 or less, where we could expect many gene effects for complex diseases to occur. Thus, despite this lack of significant association noted in this study, we believe that validation with external populations and the use of better-powered studies in the future may allow more conclusive findings.

## Background

Chronic rhinosinusitis (CRS) is a frequent and important chronic disease, which causes significant patient discomfort and morbidity. Patients with CRS report a significantly lower quality of life index in measures of bodily pain and social functioning than do patients with congestive heart failure, angina, chronic obstructive pulmonary disease, and back pain [1]. In health surveys, CRS affect more than 10% of individuals in western countries, with an overall direct cost of CRS in the United States annually estimated at $4.3 billion [2,3].

Despite the frequency of CRS and the costs associated with its care, there is limited knowledge about the initial development of disease. Our current conception of CRS is as of a chronic inflammation of the paranasal sinus membrane, which is colonised with nasal and exogenous bacteria that may contribute to the disorder. Histologically, CRS is characterized by an accumulation of inflammatory cells that are mainly eosinophils, with a neutrophilic infiltrate only during acute infections. Numerous pro-inflammatory cytokines and chemokines, both Th-1 and Th-2 profiles, are over expressed in CRS. These include IL-2, IL-4, IL-5, IL-6, IL8, IL-13 and IL-16 [4].

Current knowledge of the disease is limited to that garnered by study of biopsy samples obtained from patients affected with well-established disease, with little information available on early events or environmental factors associated with development of disease. Genetic studies may thus offer a better insight into the pathogenesis of the disorder. Recently, the primary interest in genetics has changed from simple Mendelian diseases, for which genotypes of some gene are the cause of disease, to more complex diseases, for which genotypes of some set of genes together with environmental factors merely alter the probability that an individual gets the disease, although individual factors are typically insufficient to cause the disease outright [5].

Reports of familial clustering of CRS support a genetic basis for the disease [6-8]. The high prevalence of patients with CRS in well-defined genetic disorders such as cystic fibrosis (CF) and primary cilia dyskinesia (Kartagener's syndrome) also supports the concept that mutated genes play a role in the pathogenesis of the disease [9]. However, it is suspected that CRS generally encountered in clinical practice is due to a complex interaction of multiple genes rather than the single gene anomalies as observed in CF and Kartagener's syndrome. A polygenic model for nasal polyps was recently confirmed by segregation analysis [10]. The same study also confirms that nasal polyp is a highly heritable disease with heritability coefficients above 60%. Interestingly, the quantitative contribution of genetic factors seems to be higher in young and recurrent patients [6,10]. A number of genetic association studies have been conducted to elucidate the genetic loci conferring risk of CRS with or without nasal polyposis. Significant difference in allele frequencies between patients and controls were observed for polymorphisms located in the HLA [11-14], CFTR [15], LTA [16], TGFB1 [17], IL1RN [18], and ADRB2 [19]. However, all these findings remain to be confirmed in larger populations.

**Figure 1:**
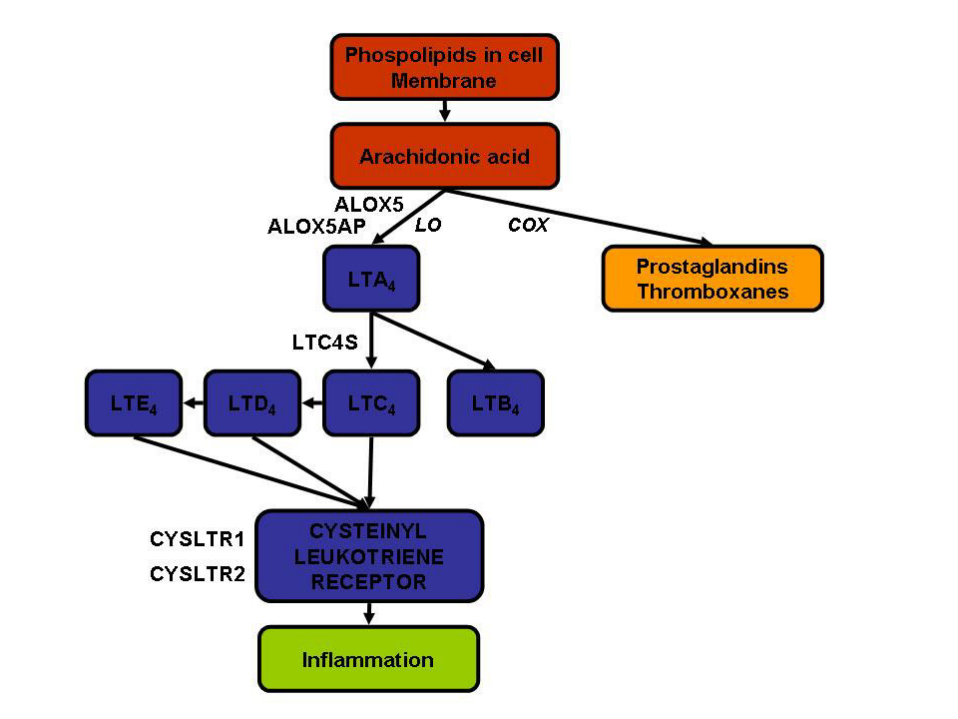
Arachidonic acid metabolism. Candidate genes selected for genotyping are indicated along the cysteinyl leukotriene metabolic pathway. LO, lipoxygenase pathway; COX, cyclo-oxygenase pathway; ALOX5, arachidonate 5-lipoxygenase; ALOX5AP, arachidonate 5-lipoxygenase-activating protein; LTC4S, leukotriene C4 synthase; CYSLTR1, cysteinyl leukotriene receptor 1; CYSLTR2, cysteinyl leukotriene receptor 2. Cysteinyl leukotriene metabolites are leukotriene B4 (LTB4), leukotriene C4 (LTC4), leukotriene D4 (LTD4), and leukotriene E4 (LTE4).

It is possible that cysteinyl leukotrienes (cysLTs) may play a disease-regulating role in rhinosinusitis. CysLTs, described primarily as lipid mediators involved in pathogenesis of airway inflammation and related symptoms such as bronchoconstriction, mucus secretion, and airway hyperresponsiveness, are recognized now as important mediators of cell trafficking and innate immune responses [20]. CysLTs are synthesized through the lipoxygenase (LO) pathway of arachidonic acid metabolism. The LO pathway for synthesizing cysLTs has several steps, and distinct enzymes are involved in each steps, namely arachidonate 5-lipoxygenase (ALOX5), arachidonate 5-lipoxygenase-activating protein (ALOX5AP or FLAP), and leukotriene C4 synthase (LTC4S) [21]. Effects at the cellular level are mediated via two G-protein coupled receptors termed cysteinyl leukotriene receptor 1 (CYSLTR1) and cysteinyl leukotriene receptor 2 (CYSLTR2) (Figure 1).

In this initial study, we propose to study the genes regulating the leukotriene pathway to identify potential polymorphisms involved in disease susceptibility. We hope that identification of genetic polymorphisms involved will lead to improved comprehension of disease pathogenesis, and may eventually lead to more effective treatment, screening, and prevention of CRS.

## Methods

### Population

210 individuals with severe CRS were prospectively recruited from three tertiary rhinology clinics from July 2005 to February 2006. Four were not included in the final analysis, as phenotypic questionnaires were incomplete, leaving 206 subjects for analysis. These were compared to 200 control subjects. The study was approved by the McGill University Health Centre ethics committee, and written informed consent was obtained from all participants.

Subjects with sever CRS were recruited from ongoing clinical activities, according to pre-established clinical criteria. Severe CRS was defined as 1) individuals with persistent signs and symptoms of CRS with one previous endoscopic sinus surgery for diagnosis of either chronic rhinosinusitis with or without sinonasal polyposis or recurrent acute sinusitis or 2) individuals with more than one surgery for these diagnoses, regardless of outcome.

A standardized questionnaire assessing age, sex, ethnic origin, and family history was obtained. Other associated factors include smoking, presence of self-reported seasonal and perennial allergies, physician diagnosed asthma and acetylsalicylic acid intolerance were also obtained. Information on disease related factors including age at diagnosis, age at first surgery, number of previous surgeries, medications and current symptoms were determined. Initial diagnoses were obtained from patients records, and classified according to 2004 American Academy of Otolaryngology-Head and Neck Surgery (AAO-HNS) guidelines [22]. Diagnosis of aspirin sensitivity was based on a medical history reporting symptoms including asthma exacerbation, rhinorrhea, and nasal congestion, after the ingestion of aspirin or other nonsteroidal anti-inflammatory drugs. Four tubes of blood were drawn from patients with severe CRS. Two were for DNA extraction and the others were for the measurements of serum eosinophilia and total IgE. Blood samples were stored at -20 degrees Celsius for 2 days then at -80 degrees Celsius prior to use.

The control population was recruited from either patient's spouse or non-blood relatives living in the same household or by random telephone screening matched to affected subject's postal code. For the 30 spouse or non-blood relative controls, whole blood was used. In the 170 control subjects recruited by telephone screening, the Oragene kit (DNA Genotek, Ottawa, Ontario) for saliva collection was sent to the control subject with prepaid return postage. Saliva samples were stored at room temperature as recommended by the manufacturer.

### IgE and eosinophilia measurements

Blood samples for total serum IgE and eosinophilia were processed by the hospital laboratory according to standard methods. Eosinophilia was reported as percent of total circulating white blood cells. IgE was reported in IU, with the upper range of normal reported as 120 IU/ml.

### DNA extraction

DNA was isolated from peripheral blood leukocytes, collected in citrate-treated tubes, using the Puregene DNA isolation kit, (Gentra System, QIAGEN) following the High Throughput Protocol for 10 ml Whole Blood provided with this kit. DNA extracted from saliva was performed using the Oragene DNA Purification Protocol (DNA Genotek, Ottawa, Ontario). Isolated DNA from both blood and saliva was stored at -20 degrees Celsius prior to use.

### SNP selection and genotyping

SNPs were selected to capture as much information as possible about genetic variation for each gene. That was achieved using the European-derived (CEU) genotype data from the International HapMap project [23] covering ten kilobases up- and downstream of each gene. From this dataset, a maximally informative set of SNPs were selected for each gene using an aggressive tagging algorithm [24] implemented in Haploview version 3.2 [25]. Minor allele frequency and r^2 ^thresholds were set at 0.05 and 0.8, respectively. SNPs selected for each gene are shown in Table 1.

**Table 1:** Association between single nucleotide polymorphisms in leukotriene pathway candidate genes and chronic rhinosinusitis.

**Gene (chr)**	**SNPs**	**MAF**	**Minor allele**	**HWE p value**	Case, Control Ratios**	**p value**
ALOX5AP (13)	rs12430915	0.08	C	0.200	38:378, 26:346	0.271
	rs4769870	0.16	T	0.080	353:61, 269:61	0.170
	rs4076128	0.30	C	0.439	290:124, 255:111	0.909
	rs11616333	0.05	G	0.558	22:396, 19:353	0.922
	rs4769055	0.33	A	0.187	143:275, 120:252	0.561
	rs9578196	0.10	T	0.408	380:38, 326:42	0.283
	rs4293222	0.37	C	0.930	161:257, 134:236	0.505
	rs12429692	0.26	T	1.000	118:300, 88:284	0.144
	rs10162089	0.49	T	0.828	217:197, 184:186	0.453
	rs4254165	0.28	C	0.928	302:114, 264:108	0.612
	rs4356336	0.42	C	1.000	242:176, 208:154	0.902
	rs17612127	0.08	T	0.847	40:376, 21:343	**0.046**
	rs9506352	0.34	T	0.300	278:138, 242:128	0.674
	rs9579648	0.15	C	0.317	68:350, 52:320	0.371
	rs9579649	0.07	T	0.330	28:388, 24:348	0.875
	rs9315051	0.08	C	1.000	33:385, 26:346	0.629
	rs4420371	0.25	C	0.842	107:311, 93:277	0.882
	rs4466940	0.20	T	0.295	85:329, 60:256	0.604
	rs9578200	0.17	T	0.014	352:66, 307:65	0.525
	rs9285076	0.22	T	0.133	326:92, 289:83	0.919
	rs9670198	0.04	A	0.168	401:15, 329:13	0.887
	rs4319601	0.41	T	0.064	241:163, 209:143	0.938
	rs4769063	0.13	T	1.000	365:51, 322:50	0.621
	rs4238139	0.28	C	0.028	117:301, 99:265	0.805
ALOX5 (10)	rs3824612	0.38	T	0.263	273:145, 220:152	0.074
	rs3780894	0.17	C	0.789	85:333, 52:320	**0.019**
	rs7099684	0.19	A	0.456	82:332, 65:301	0.466
	rs7919239	0.23	A	0.392	101:313, 79:293	0.293
	rs2115819	0.47	C	0.414	195:219, 152:180	0.720
	rs11239523	0.16	C	0.831	71:345, 55:317	0.383
	rs4948672	0.46	C	0.512	203:215, 161:211	0.137
	rs12264801	0.45	T	0.028	230:188, 202:168	0.904
	rs3780901	0.33	C	0.440	143:271, 116:250	0.400
	rs2279435	0.43	C	0.707	187:231, 156:216	0.428
	rs1565096	0.23	C	0.094	323:95, 283:89	0.691
	rs1487562	0.21	T	0.381	97:321, 69:301	0.117
	rs2291427	0.30	T	0.870	298:120, 257:115	0.498
	rs7393696	0.38	A	0.394	256:154, 207:135	0.591
	rs7089063	0.24	A	0.478	112:304, 61:233	0.059
CYSLTR2 (13)	rs2406939	0.36	C	0.948	152:266, 130:242	0.678
	rs11617224	0.11	C	1.000	375:43, 329:41	0.719
	rs6420296	0.08	C	1.000	389:29, 337:33	0.303
	rs7335898	0.06	G	0.790	28:390, 18:336	0.345
	rs9285169	0.09	T	0.136	43:373, 26:334	0.129
	rs9595961	0.48	C	0.994	203:215, 175:195	0.722
	rs17072059	0.04	T	0.784	407:11, 353:17	0.137
	rs7330127	0.44	A	1.000	189:229, 155:207	0.501
	rs2407249	0.21	C	0.129	88:330, 74:298	0.687
	rs9568087	0.29	A	0.315	299:117, 254:108	0.600
	rs12184704	0.07	C	0.057	33:385, 21:339	0.259
CYSLTR1 (X)*	rs321090	0.23	C	0.605	247:60, 207:81	**0.014**
	rs321007	0.30	C	0.916	224:84, 192:96	0.107
	rs321006	0.13	T	0.423	42:266, 37:252	0.764
LTC4S (5)	rs730012	0.29	C	0.403	126:292, 103:269	0.448
	rs2291418	0.04	T	0.316	399:15, 347:19	0.284
	rs166624	0.17	A	1.000	77:341, 56:316	0.207

HWE, Hardy-Weinberg equilibrium; MAF, minor allele frequency.

*Hardy-Weinberg and association tests were specifically calculated for the X chromosome using the method implemented in Haploview 3.32.

**Number of allele A observed in cases : number of allele B observed in cases, number of allele A observed in controls : number of allele B observed in controls.

SNPs were genotyped using Sequenom matrix-assisted laser desorption/ionization time-of-flight (MALDI-TOF) mass array spectrometer (Sequenom, San Diego, CA). Primers were designed using the Sequenom SNP Assay Design software version 3.0 for iPLEX reactions. A total of 59 assays were designed within a 29-, 24- and 7-plexs reactions. The protocol and reaction conditions were in accordance with the manufacturer [26].

### Statistical analysis

Sample size was designed to provide 95% power to detect common alleles (> 10%) conferring a > 3.0 -fold increase in risk, and 50% power to detect alleles >25% that increase risk by a factor of 2.0.

Hardy-Weinberg equilibrium (HWE) was estimated using a χ^2 ^test. HWE tests were performed only in females for SNPs located on the X chromosome (SNPs located in the CYSLTR1 gene). Case-control comparisons were analyzed using a χ^2 ^test implemented in Haploview version 3.2 [25]. A nominal p-value less than 0.0012 was considered significant. This threshold was computed by permutation testing to ensure a project-wide type 1 error rate of 0.05.

## Results

210 patients with severe CRS were recruited. Four were not included in the analysis, as phenotypic questionnaires were incomplete. Table 2 shows the characteristics of cases and controls. For cases, initial diagnosis was recurrent rhinosinusitis in 12.1%, CRS without polyposis in 13.1%, and CRS with polyposis in 74.8%. Average number of previous surgeries was 3.15 (range 1 – 20; median 2.0), with an average age at first surgery of 38.3 years (range 1 – 76; median 38). Medication use in this group was high, with 73.4% of patients requiring medication beyond an inhaled nasal corticosteroid for disease control and 23.3% remaining uncontrolled despite medication. Atopy was present in 65.5% and asthma requiring treatment in 53.9%. Active smoking was present in 11.2%. Patients reported blood relatives affected with CRS in 37.7% of instances.

**Table 2:** Characteristics of the subjects

	**Cases**	**Controls**
n	206	200
Age (years)	52.3 ± 13.0	48.8 ± 15.0
Male: Female ratio	1.10	0.79
Ethnic group (%)		
White	85.2	89.5
Middle East	4.9	4.4
Jewish	5.4	1.1
Asian	1.5	1.1
First Nation	1.5	0.6
Black	1.0	0.6
Hispanic	0.5	1.1
Pacific Islander	0.0	1.7

Values are means ± SD for age.

Measured serum biomarkers showed mean circulating eosinophilia of 4.4% (range 0 – 25%; median 4.0%) with 33.5% of patients demonstrating > 5% eosinophilia. Mean total serum IgE was 155.9 IU (range 2 – 1460, median = 85.0) with 41.1% having levels IgE > 120 IU/ml.

### Genetic analysis

The assay conversion rate was 95% (56 out of 59) and the average genotyping success rate for the 56 SNPs yielding working assays was 98.7%. The remaining 3 SNPs (rs4503649, rs12561364 and rs1359112) failed the genotyping assay and were discarded from the analyses. All SNPs were in HWE, except rs9578200 where the p-value was 0.014 (Table 1).

**Figure 2:**
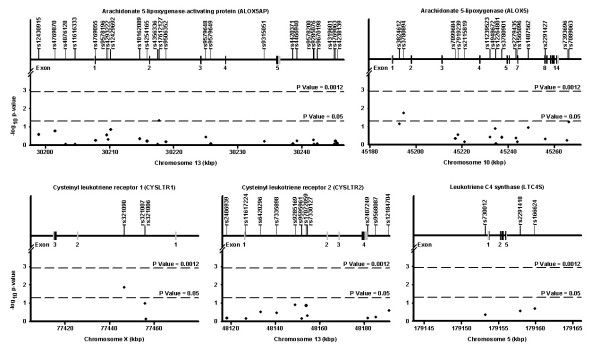
Genetic association of SNPs in the leukotriene pathway genes with chronic rhinosinusitis. Each subfigure presents the result of one gene. The top line indicates the gene name and symbol. The upper part of each subfigure shows the exon-intron structure of the gene and the localization of the genotyped SNPs. The coding exons are shown in black and the untranslated regions are shown in grey. The lower part of each subfigure illustrated the association results for chronic rhinosinusitis. The x-axis shows the localization of the gene and SNPs on build 36. The y-axis shows the p values on a log scale. The lower and upper dash lines represent p value thresholds of 0.05 and 0.0012, respectively. The gene structure for CYSLTR2 was taken from Fukai et al. [38]. The upper and lower parts of each subfigure are shown on the same scale.

**Figure 3:**
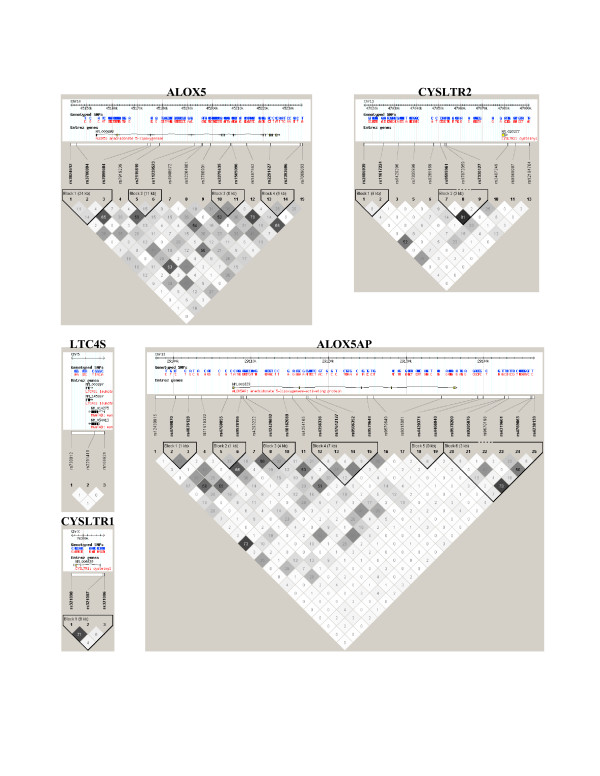
Linkage disequilibrium (LD) plots surrounding genes involved in the leukotriene pathway. The LD plots were generated by Haploview 3.32. [25]. Gene symbols are indicated at the top of each graph. The upper part of each graph illustrated the HapMap info track including the location of the gene on build 34. The white horizontal bar below the info track illustrated the location of SNPs on a physical scale. The color of squares illustrate the strength of pairwise r^2 ^values on a black and white scale where black indicates perfect LD (r^2 ^= 1.00) and white indicates perfect equilibrium (r^2 ^= 0). The r^2 ^LD value is also indicated within each square. Blocks are defined using the Gabriel et al. [39] definition. Failed and monomorphic SNPs as well as SNPs not in Hardy-Weinberg equilibrium are not illustrated.

A final collection of 56 SNPs was tested for association. Figure 2 illustrates the location of each SNPs relative to the intron-exon structure of each gene. Figure 2 also depicts the genetic association results between SNPs and CRS. Three SNPs were found to be associated with CRS at a p value < 0.05. These SNPs were found in the ALOX5 (rs3780894), ALOX5AP (rs17612127) and CYSTLR1 (rs321090) genes. However, none of these SNPs reached the multiple testing p value threshold of 0.0012. The linkage disequilibrium pattern for each gene in our population is illustrated in Figure 3.

Spurious associations cause by population stratification is always a concern in genetic case-control association studies. Accordingly, the analyses were repeated with only White individuals (162 controls and 173 cases). Globally the results were very similar. All SNPs were in HWE and the minor allele frequencies were practically unchanged compared to all ethnicities included. The SNPs ALOX5-rs3780894 and CYSLTR1-rs321090 remained statistically significant (p = 0.037 and 0.031, respectively). In contrast, SNP ALOX5AP-rs17612127 was no longer significant (p = 0.144).

## Discussion

In this study, we have analyzed SNPs covering 5 genes implicated in the cysLT pathway in a case-control series of patients with severe CRS. After adjustment of multiple testing, there were no significant associations observed between severe CRS and genetic variants within these genes. Rather than suggest that there is no role for polymorphisms in the leukotriene cascade, it suggests that the effect of polymorphisms in cysLTs is less than the power detectable by our population size, which is well-powered to detect only polymorphisms in common alleles which confer a relative risk (RR) of 3.0 or greater, and only moderately powered to detect polymorphisms with a RR of 2.0. In this study, the minor allele of the most significant polymorphisms conferred a RR of 1.45 (ALOX5-rs3780894), 1.67 (ALOX5AP-rs17612127) and 0.69 (CYSLTR1-rs321090). Most genes for complex diseases that have been described have an effect in this range or less.

Although SNP CYSLTR1-rs321090 is not significant after multiple testing correction, the results observed in the current study replicated the previous association reported by Hao et al [27]. In fact, the polymorphism, labeled 927T/C (rs320995) was significantly associated with atopy severity in a group of asthmatic families. Interestingly, this polymorphism is in tight linkage disequilibrium (LD) with rs321090 (r^2 ^= 0.82 based on the CEU genotyping dataset of the International HapMap project [23]). In both studies, the common allele is more frequent in cases than controls. In contrast, the current study does not corroborate the previous association between SNP LTC4S-rs730012 (also known as -444A/C) and allergic rhinitis [28]. However, a number of controversies exist concerning the role of this polymorphism in allergic-related diseases [29,30]. Accordingly, larger and well-powered studies will be required to determine the role of this polymorphism in pathogenesis of these diseases.

Beside the lack of power, other factors may explain the absence of significant results. First, we have studied what is a relatively heterogeneous population, with several different initial diagnoses, some unresponsive to therapy effective in others and different levels of serum biomarkers of inflammation. It is possible that the effect of cysLTs may be different within these groups and less noticeable in the group as a whole. Analysis of subgroups within our population may be of benefit but will be limited by sample size. Secondly, the control group utilized must also be considered. The group used has been selected for comparability in terms of environment rather than for absence of the disease. With the prevalence of self-reported chronic sinusitis being reported as high a 16% and the incidence of allergies being over 20%, confusion with these other disorders may make it hard to differentiate from these other disorders.

Leukotrienes have nevertheless been implicated in CRS in other models and several studies have shown that leukotrienes may be involved in the development of the disorder. CysLT metabolites, leukotriene C4 (LTC4), leukotriene D4 (LTD4), and leukotriene E4 (LTE4) likely contribute to the pathogenesis of chronic rhinosinusitis through their effects on microvascular leakage, epithelial cell activation, elevated mucus secretion, and mucosal inflammation [31]. CysLTs also appear to be related to the severity of eosinophilic inflammation [32].

CysLTs appear related to nasal polyposis. In an examination of 27 patients by Kaplan et al. [33] patients with sinonasal polyps were noted by radioimmunoassay to have elevated levels of LTC4. Patients with sinonasal polyposis with recurrences within 18 months of surgery had higher levels of LTC4 when compared with those patients that did not have recurrences. In another study of 58 individuals, Steinke et al. [34] reported that chronic hyperplastic eosinophilic sinusitis is characterized by the increased presence of CysLTs when compared with concentrations seen in tissue from patients with chronic inflammatory sinusitis or healthy sinus tissue. Specifically implicating the polyp tissue as the source of these cysLTs, Higashi et al. [35] found that there were significant decreases in the urinary LTE4 concentrations before and after the sinus surgery for patients with chronic hyperplastic rhinosinusitis with nasal polyposis. In support of this, a recent study showed that urinary LTE4 concentration is highly significant in 10 patients with aspirin-intolerant asthma (AIA) with nasal polyps compare to patients with 24 AIA without polyps [36].

Despite the evidence supporting a role for leukotrienes other authors have failed to demonstrate a role for cysLTs in CRS. A study by Kountakis et al [37] showed that mean cysLTs levels were similar in CRS patients with eosinophilic versus non eosinophilic and in patients with polyps versus without polyp. Also cysLTs levels did not correlate with severity of CRS according to CT findings, endoscopy findings, or symptom scores.

## Conclusion

While these initial results do not support that polymorphisms in genes assessed involved in the leukotriene pathways are contributing to the pathogenesis of CRS, this initial study was not powered to detect polymorphisms with relative risk of 2.0 or less, where we could expect many gene effects for complex diseases to occur. Thus, despite this lack of significant association noted in this study, we believe that validation with external populations and the use of better-powered studies in the future may allow more conclusive findings.

## List of abbreviations

AIA: aspirin-intolerant asthma, ALOX5: arachidonate 5-lipoxygenase, ALOX5AP: arachidonate 5-lipoxygenase-activating protein, CF: cystic fibrosis, CRS: Chronic rhinosinusitis, COX: cyclo-oxygenase pathway, CYSLTR1: cysteinyl leukotriene receptor 1, CYSLTR2: cysteinyl leukotriene receptor 2, cysLTs: cysteinyl leukotrienes, HWE: Hardy-Weinberg equilibrium, LD: Linkage disequilibrium, LO: lipoxygenase pathway, LTB4: leukotriene B4, LTC4: leukotriene C4, LTC4S: leukotriene C4 synthase, LTE4: leukotriene E4, RR: relative risk, SNP: single nucleotide polymorphism

## Competing interests

The author(s) declare that they have no competing interests.

## Authors' contributions

HAS was involved in sample collection and draft the first version of the manuscript. YB carried out SNP selection and genotyping, performed statistical analyses, and finalized the manuscript. TJH participated in the design of the study. MC, MD, SVS, SF were involved in sample collection and coordination of the case-control population. ML calculated the p-value threshold to ensure a project-wide type 1 error rate of 0.05. MD conceived the study, acquired the funding, and provided general supervision of the research group. All authors read and approved the final manuscript.

## Pre-publication history

The pre-publication history for this paper can be accessed here:


